# Burden of disease among older adults in Europe—trends in mortality and disability, 1990–2019

**DOI:** 10.1093/eurpub/ckac160

**Published:** 2022-11-24

**Authors:** Kim Moesgaard Iburg, Periklis Charalampous, Peter Allebeck, Elsa Jonsson Stenberg, Rónán O’Caoimh, Lorenzo Monasta, José L Peñalvo, David M Pereira, Grant M A Wyper, Vikram Niranjan, Brecht Devleesschauwer, Juanita Haagsma

**Affiliations:** Department of Public Health, Aarhus University, Aarhus, Denmark; Department of Public Health, Erasmus MC, University Medical Center Rotterdam, Rotterdam, The Netherlands; Department of Global Public Health, Karolinska Institute, Stockholm, Sweden; Department of Global Public Health, Karolinska Institute, Stockholm, Sweden; Department of Geriatric and Stroke Medicine, Mercy University Hospital and University College Cork, Cork, Ireland; Clinical Epidemiology and Public Health Research Unit, Institute for Maternal and Child Health—IRCCS Burlo Garofolo, Trieste, Italy; Unit of Non-Communicable Diseases, Department of Public Health, Institute of Tropical Medicine, Antwerp, Belgium; REQUIMTE/LAQV, Laboratório de Farmacognosia, Departamento de Química, Faculdade de Farmácia, Universidade do Porto, Porto, Portugal; School of Health & Wellbeing, University of Glasgow, Glasgow, UK; School of Public Health, Physiotherapy and Sport Sciences at University College Dublin, Dublin, Ireland; Department of Epidemiology and Public Health, Sciensano, Brussels, Belgium; Department of Translational Physiology, Infectiology and Public Health, Ghent University, Merelbeke, Belgium; Department of Public Health, Erasmus MC, University Medical Center Rotterdam, Rotterdam, The Netherlands

## Abstract

**Background:**

It is important to understand the effects of population ageing on disease burden and explore conditions that drive poor health in later life to prevent or manage these. We examined the development of disease burden and its components for major disease groups among older adults in Europe over the last 30 years.

**Methods:**

Using data from the Global Burden of Disease 2019 Study, we analyzed burden of disease trends between 1990 and 2019 measured by years of life lost (YLL), years lived with disability (YLD) and disability-adjusted life years (DALYs) among older adults (65+ years) in Western, Central and Eastern Europe using cause groups for diseases and injuries.

**Results:**

Between 1990 and 2019, the crude numbers of DALYs for all causes increased substantially among older Western Europeans. In Eastern Europe, the absolute DALYs also increased from 1990 to 2005 but then decreased between 2006 and 2013. However, DALY rates declined for all European regions over time, with large differences in the magnitude by region and gender. Changes in the YLL rate were mainly driven by the contribution of cardiovascular diseases.

**Conclusions:**

This study found an increased overall absolute disease burden among older Europeans between 1990 and 2019. The demographic change that has taken place in Eastern European countries implies a potential problem of directed resource allocation to the health care sector. Furthermore, the findings highlight the potential health gains through directing resources to health promotion and treatment to reduce YLDs and to prevent YLLs, primarily from cardiovascular diseases.

## Introduction

The increase in life expectancy observed in almost all countries, while a success, is also recognized by policymakers and health professionals as a public health challenge. Increased longevity often implies additional disease burden from age-related disease and increasing dependency ratios. A recent population projection underlined the fact that population ageing will continue for the next couple of decades in Europe with an expanding number of older people and shrinking proportions of people of working age, as the post-war baby-boom generation completes its move into retirement.^1^ This increased life expectancy is not only driven by reduced mortality in infants and young children, but also by increased survival among older people.^2^ Paradoxically, better health is the main driver of increased longevity, but living longer is often associated with additional health problems. To what extent increased life expectancy leads to a rise in morbidity over time, or compression of morbidity into later in the lifespan, is a long-standing debate.^3^ There is evidence for both better health and well-being, but also increasing morbidity and greater care needs among older people.^4^ However, the absolute measure of disease burden is often conflated with the relative, and age-standardized, disease burden. It has been evidenced that age-standardized, age-related disease burden is decreasing globally and the main proportion of disease burden among older people is driven by general population growth, which varies across different parts of the world.^5^

From a public health perspective, it is important to understand the socio-economic and environmental determinants of disease burden, and to gather knowledge about the main disorders that drive poor health and that need to be prevented or treated. For prioritization in public health policy and for logistic planning purposes, the absolute level of disease burden is important to know. Even if the relative and age-standardized disease burden declines, due to improved health and treatments, population growth and ageing still imply that the absolute level of disease burden increases, and maybe considerably so, such that the additional pressures generated on health and social services cannot be met, without considerable bolstering of resources in terms of availability and staffing.

The Global Burden of Disease (GBD) study provides methodological concepts and data to study disease burden considering both premature mortality [years of life lost (YLL)] and morbidity [years lived with disability (YLD)], providing updated estimates on their website (http://ghdx.healthdata.org/). Using these data, Chang et al.^5^ provided a comprehensive analysis of age-related disease burden globally and over time using GBD 2017. The authors observed an overall decline in age-standardized disease burden but with considerable variation across countries. In this article, we focus on characterizing ageing in Europe.

There are several public health and policy relevant research questions related to ageing in Europe. Firstly, there are marked differences in life expectancy, fertility and migration in different areas of Europe.^6^ Several Eastern European countries are characterized by considerable emigration and low fertility, whereas the opposite is observed in some Western European countries. It is important to understand how this affects differences in disease burden. Economic effects have already been reported for the East–West migration on economic outcomes in the European Union and Eastern Partnership countries.^7^

Secondly, most European countries have built up systems for universal coverage of health and social services since the middle of the 20th century. There is concern that current services are struggling to sustain the needs of an ageing population, so health systems need to be reviewed and age-attuned.^8^^,^^9^

Thirdly, the funding and provision of pension schemes developed during the 20th century are under review in most European countries with the likelihood that many individuals will continue to work beyond traditional retirement ages, e.g. 65 years.^10^ To understand disease burden and how it has developed across older age-groups, the debate around retirement is important, although many other factors also determine the ability to work across the life course.^11^^,^^12^ Results from the European Community Household Panel survey showed that Europe is facing demographic ageing just as life expectancies are increasing and birth rates decreasing while at the same time average retirement ages have been decreasing.^13^

Against this background, we have examined the drivers of burden of disease in Western, Central and Eastern Europe during the period 1990–2019, using YLL and YLD. Specifically, we have investigated:

- How disease burden in older adults has changed over time, both in absolute terms—and considering population growth and ageing
- How disease burden is distributed by age, in older adults
- What conditions contribute to the main components of disease burden in older age, and how they changed over time
- What differences in disease burden are found across different European regions and how this has changed over time

## Methods

We performed a secondary analysis of the GBD 2019 study, analyzing burden of disease trends. Burden was measured using YLL, YLD and disability-adjusted life years (DALYs) among older adults (65+ years) in Europe, during the period from 1990 to 2019. YLL is calculated by multiplying deaths by the remaining life expectancy at the age of death from an aspirational standard life table developed for the GBD study. YLD is calculated by multiplying prevalence of disease by the corresponding disability weight assigned to defined health states, and adjusted for comorbidity. DALYs are calculated by adding YLL and YLD.

The GBD 2019 study provides internally consistent and comparative estimates of prevalence, incidence, mortality, YLL, YLD and DALY by cause, age, sex, year and geographical locations for the years 1990–2019. Detailed descriptions of the methodological approaches of the GBD 2019 are found elsewhere.^14^ For this study, we retrieved both crude numbers and YLL, YLD and DALY rates per 100 000 by sex and age-groups over 65 years for the European regions of the GBD by 5-year periods in the 1990–2019 timeframe. All data were extracted through the GBD Results tool (http://ghdx.healthdata.org/gbd-results-tool).

### Hierarchy of disease causes

In GBD 2019, diseases and injuries were aggregated into a hierarchy, starting from the broadest Level 1 to a detailed Level 4. Level 1 composed of three overall groups. Group I: communicable, maternal, neonatal and nutritional diseases; Group II: non-communicable diseases (NCDs); and Group III: injuries. These are further subdivided into 22 Level 2 causes, 174 Level 3 causes and 301 Level 4 causes. For the present analysis, we report on the burden of disease by Level 2 causes.

### Countries of the European regions

We report disease burden according to the three European regions defined in GBD 2019: Western, Central and Eastern Europe. The following 24 countries are included in the GBD Western European region: Andorra, Austria, Belgium, Cyprus, Denmark, Finland, France, Germany, Greece, Iceland, Ireland, Israel, Italy, Luxembourg, Malta, Monaco, the Netherlands, Norway, Portugal, San Marino, Spain, Sweden, Switzerland and UK. The following 13 countries are included in the GBD Central European region: Albania, Bosnia and Herzegovina, Bulgaria, Croatia, Czechia, Hungary, Montenegro, North Macedonia, Poland, Romania, Serbia, Slovakia and Slovenia. The following seven countries were included in the GBD Eastern European region: Belarus, Estonia, Latvia, Lithuania, the Republic of Moldova, the Russian Federation and Ukraine. For the present analysis, we report on the burden of disease by European regions. Burden of disease by country within these regions is not reported.

### Age and population adjustments

We extracted absolute numbers, rates per 100 000 population, and age-adjusted rates per 100 000, overall and stratified by sex, year, location and 10-year interval age-groups (i.e. 65–74; 75–84; 85–94; and 95+) from GBD 2019. Absolute numbers are defined as the total number of YLLs, YLDs or DALYs during a given year, and incorporate how the epidemiological burden of disease has changed in relation to changes in both the demographic and epidemiological context. Routine age-standardized rates in GBD 2019 are estimated using the GBD World Standard Population (WSP) structure; age-specific distributions of all national locations are drawn from the World Population Prospects 2012 version and generate a GBD standard structure for the whole population of all ages by taking the non-weighted mean across locations and years.^15^ However, we calculated our own age-adjusted rates using the European Standard Population (ESP) since this standard population is more appropriate for European regions, and it has been evidenced that the use of the WSP in the wrong context could lead to quite different conclusions.^16^ Age-adjusted rates are defined as YLL, YLD or DALY rates that would have occurred if the observed rates were present in each population where the age-distribution is that of the standard population (e.g. ESP), and are key indicators when describing how the epidemiological burden of disease has changed.

Disease burden estimates for the age-group 85–94 years is not a standard option in the GBD Results tool. Therefore, we summed YLLs, YLDs or DALYs for those aged 85–89 and 90–94 years into one group: ages 85–94 years. As a consequence, we are not able to calculate uncertainty intervals (UI) for the estimates, we present. To calculate the total absolute number for the 85–94 group, we summarized the crude numbers of the 85–89 and 90–94 age-groups, for each specific cause of disease, sex, location and year. To calculate rates per 100 000 of the population, we divided the total crude number of those aged 85–94 years by the total population of this age-group category. The sums were then multiplied by 100 000. To sum the rates across older ages to obtain all-age (65+), we divided the total DALYs by the total population. Age-adjusted rates across older age-groups were calculated by multiplying age-specific rates by the latest ESP weights for 2013^17^ for each age-group.

### Data processing

Firstly, we examined disease burden as:^1^ absolute number of DALYs;^2^ DALYs per 100 000; and^3^ age-adjusted DALYs per 100 000 in Western, Eastern and Central Europe, from 1990 to 2019. Secondly, we examined the distribution of YLL and YLD age-adjusted rates per 100 000 for the top-10 Level 2 causes for males and females across older age-group categories and location over the 30-year study period. Thirdly, we analyzed DALY rates per 100 000 of population related to all Level 2 causes, age-group categories, for both males and females in the three European regions of the GBD for the 1990 and 2019 years.

## Results

### Trends of all-cause DALYs in Europe, 1990–2019

**Figure 1:**
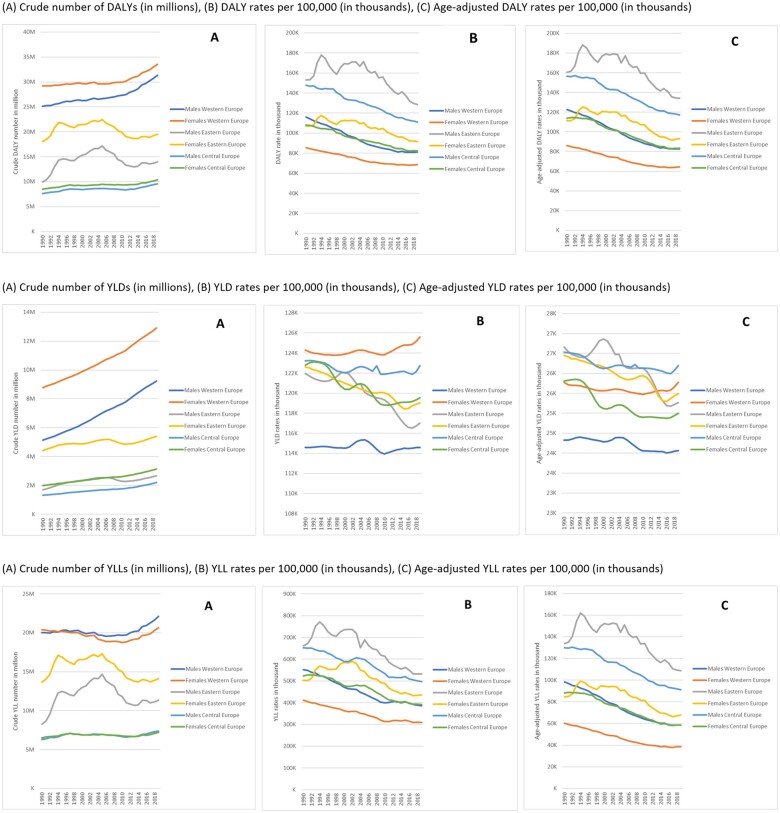
Total all-cause DALYs in Western, Eastern and Central Europe, 1990–2019. (A) Crude number of DALYs (in millions), (B) DALY rates per 100 000 (in thousands) and (C) age-adjusted DALY rates per 100 000 (in thousands)

Figure 1 shows the total all-cause DALYs in absolute numbers, rates and age-adjusted rates in Western, Eastern and Central Europe over the last 30 years. Overall, the crude number of DALYs was higher among females than males in all European regions with the biggest difference found in Eastern Europe. Between 1990 and 2019, the crude number of DALYs due to all Level 2 causes increased among Western Europeans aged ≥65 years of age, particularly from 2010 onwards. In Eastern Europe, the crude number increased between 1990 and 2005 and then decreased between 2006 and 2013. Meanwhile, in Central Europe, the crude number of all-cause DALYs was lower compared to Western and Eastern Europe.

Overall, DALY rates and age-adjusted DALYs per 100 000 among older adults in all European regions declined substantially (figure 1). Eastern European females showed a similar disease burden profile to that of Central European females and Western European males. Trends in all-cause DALY rates and age-adjusted DALYs for older adults varied, from lowest rate in females in Western Europe to highest rate in males in Eastern Europe.

Between 1990 and 2019, the percentage change among older males decreased substantially in DALY rates (−30% in West, −16% in East and −25% in Central) as well as age-adjusted DALYs (−33% in West, −16% in East and −25% in Central). DALY rates also decreased for females (−20% in West, −14% in East and −24% in Central). The same trend was seen among females when DALYs were age-adjusted (−25% in West, −16% in East and −27% in Central).

The decomposition of DALYs to YLD and YLL rates are also shown in figure 1. YLD rates are markedly different when not adjusted for age, especially in Western Europe, which shows very high levels, increasing since 2010 for females.

### Trends of age-adjusted YLLs and YLDs in Europe, 1990–2019

**Figure 2:**
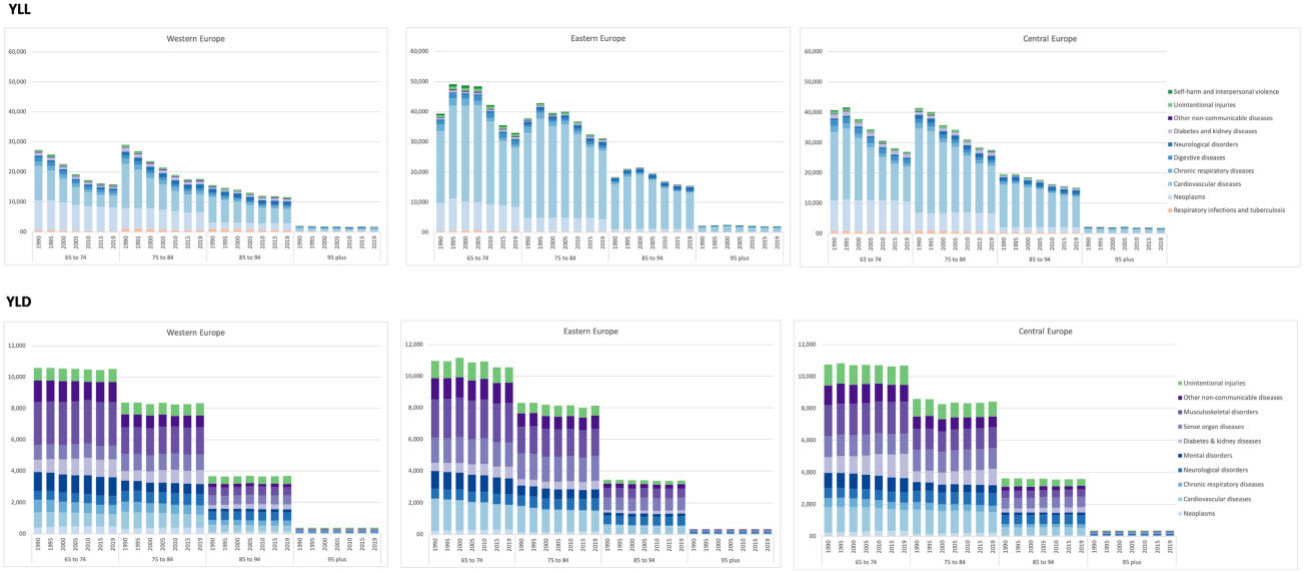
Age-adjusted YLL and YLD rates for top-10 Level 2 cause of diseases, by age-group, in both sexes in Western, Eastern and Central Europe, 1990–2019

Figure 2 shows the age-adjusted YLL and YLD rates for the top-10 Level 2 causes by age-group, for both sexes in Western, Eastern and Central Europe between 1990 and 2019. The YLD rates remained remarkably similar over the period between 1990 and 2019 and the reduction by age is very similar across all European regions.

YLL rates in Eastern Europe increased during the 1995–2005 period and then declined from 2006, creating an inverted U-shape with a peak around 2000, which was most obvious in the 64–74 and 75–84 age-groups. In Western and Central Europe, there was a very marked decline in YLL in the 65–74 and 75–84 age-groups.

The change in the YLL rate was mainly driven by changes in cardiovascular diseases (CVDs), see figure 2, including the increase and subsequent decrease observed in Eastern Europe.

Disease burden due to YLD rates was driven by a similar distribution of disease categories across the three European regions and with very similar decline by age-group. Between 1990 and 2019, YLD rates due to diabetes and kidney diseases increased among older European adults. YLDs caused by neurological disorders also increased by age in all European regions. A small decline in age-adjusted YLDs due to mental disorders was observed across all older age-groups and regions.

### Burden of diseases in European regions, 1990 and 2019

**Figure 3:**
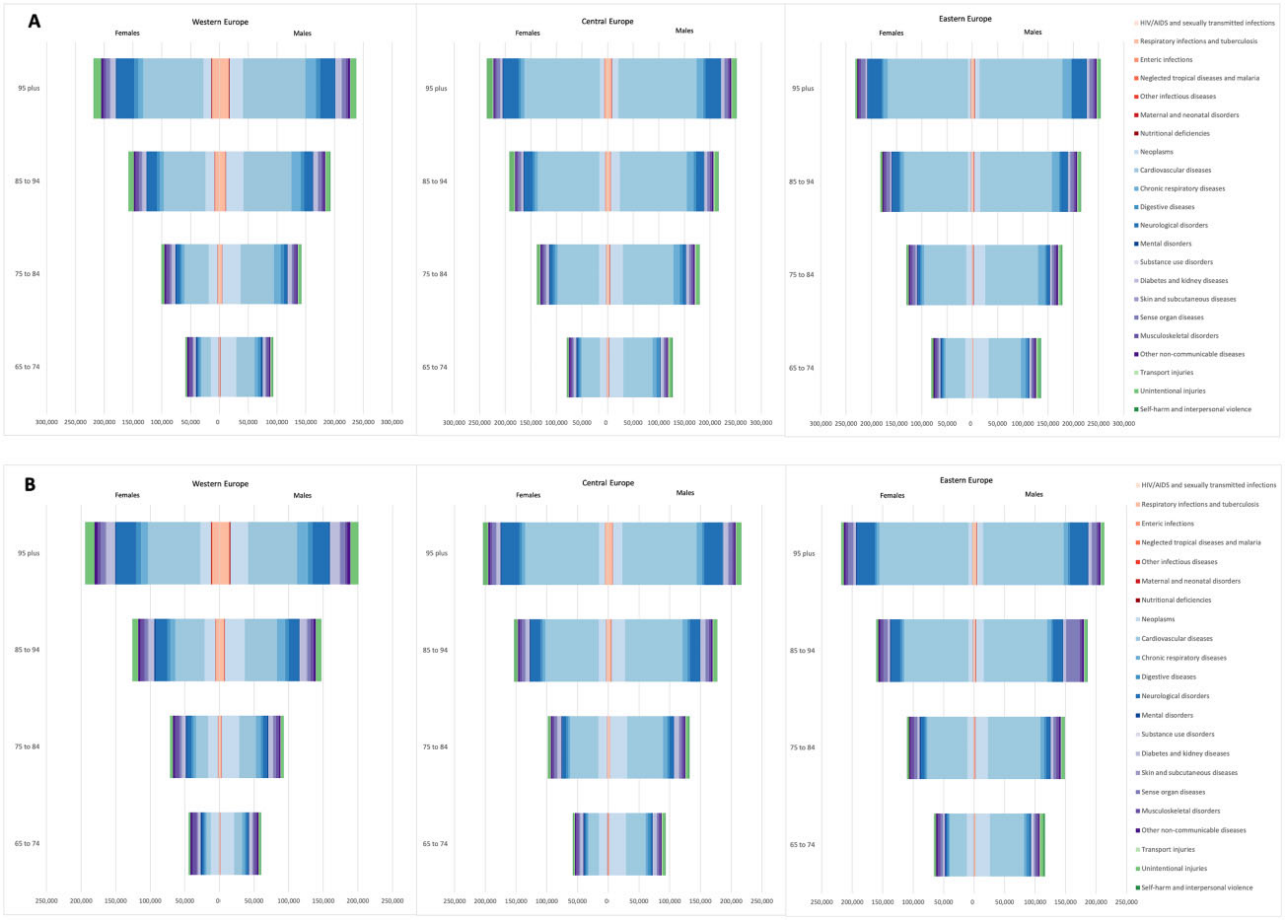
DALY rates per 100 000 by Level 2 cause of diseases, age-group and sex in Western, Central and Eastern Europe, (A) 1990 and (B) 2019

Figure 3 shows the DALY rates per 100 000 by Level 2 cause of diseases, age-group and sex in Western, Central and Eastern Europe in 1990 and 2019. In 1990, crude DALYs per 100 000 were lowest in older adults in Western Europe, while they were similar in older persons across Eastern and Central Europe. In all regions, CVDs, neoplasms and neurological disorders were the three leading causes of NCD burden. Additionally, DALYs due to CVDs, neoplasms, chronic respiratory diseases and substance use disorders were found to be higher in older European males than in females. Specifically, in 1990, the latter two causes of DALYs were two times higher in older males than in females across all European regions. Contrary to this, the disease burden of mental disorders was identified as higher in older females than in males. In 1990, DALYs caused by diabetes and kidney diseases were higher in older males (West: 26 405 DALYs per 100 000; East: 10 010 DALYs per 100 000; Central: 19 780 DALYs per 100 000) compared to females (West: 23 475 DALYs per 100 000; East: 8367 DALYs per 100 000; Central: 17 629 DALYs per 100 000). Also, the burden of unintentional injuries among Western and Central European females aged between 85–94 and 95+ was higher than in Western and Central European males (85–94 and 95+), while in Eastern Europe, DALYs of unintentional injuries were ∼2 times higher in males compared to females across the age-groups.

In 2019, crude DALYs were lowest in Western Europe and highest in Eastern Europe. The burden of disease increased with age in both males and females across the three European regions. In Europe, CVD DALY rates for males aged 65–74 were approximately double those for females of the same age (West: 11 820 DALYs in males vs. 5891 DALYs in females per 100 000; East: 55 998 DALYs in males vs. 28 506 DALYs in females per 100 000; and Central: 31 773 DALYs in males vs. 17 297 DALYs in females per 100 000). Across all three European regions, crude DALYs due to neoplasms and diabetes were higher in older males than in females, while musculoskeletal and neurological disorders accounted for greater DALYs in older European females. Older adults in Western Europe generally had higher DALYs for unintentional injuries compared to the rest of Europe. Moreover, DALYs due to self-harm and interpersonal violence among females in Eastern Europe (1663 DALYs per 100 000) were almost two times higher compared to females in Western (652 DALYs per 100 000) and Central Europe (831 DALYs per 100 000).

## Discussion

We found a significant increase in the overall absolute disease burden among persons aged 65 and above in Western Europe, especially during the last decade. Other European regions had a less pronounced increase and even a reduction in the disease burden in Eastern Europe since the 2000. Our analysis shows that these differences were mainly driven by changes in population size and ageing patterns. There was an overall reduction in the age-adjusted disease burden in all three European regions. The greatest reduction was among males in Western Europe and females in Central Europe, whereas the smallest reduction was found in the Eastern European region, among both females and males. The demographic and epidemiological transition that have taken place in Eastern European countries may suggest a potential problem of resource allocation to the health care sector and more concretely a current lack of staff in the service sector. More generally, our findings have important consequences for the future design of healthcare workforces and services. The slowing of ageing progress in epidemiological trends, reported for many countries, has worrying implications for the future in terms of adding economic, social and health related burden. Our findings highlight areas for which older adults are encountering the largest health losses, which provides insights into where preventive and mitigation strategies may be best placed. Understanding these calculations would help to plan public health and health promotion interventions and policies for this specific age-group.

A reduction in age-adjusted disease burden over time has also been shown globally,^5^ although differences in Europe have not been highlighted to date. Here, we found that the distribution by YLL and YLD was very different between the three European regions examined. In Western Europe, the age-adjusted level of YLL and YLD was at a rather equal level as components of the total burden of disease, whereas in the Central and Eastern regions, the share of YLL was much higher than YLD. Also, the decline in disease burden over the last 30 years was mainly driven by a reduction in YLL. This could suggest the potential for Central and Eastern European countries to reduce disease burden by directing resources to health promotion and early intervention to prevent premature deaths from CVDs primarily, and other NCDs, as the major components of the burden.

The rate of YLDs has not declined in the three European regions over the last 30 years; we found that only small improvements have taken place. With more people living longer, we would have expected YLD to grow, but instead YLDs have remained constant, with a shift to older age-groups. This may also point towards the potential for future improvement. European countries have developed increasingly evidence-based prevention programmes and advanced health care capable of preventing or managing conditions for many older Europeans. Although studies have shown a seemingly successful ageing process with increasing levels of physical, mental and social well-being and more active lives amongst older people,^18^^,^^19^ the results here and the distribution of components of disease burden give cause for caution. Perhaps while life expectancy has increased in all three European regions the last 30 years, the overall health status may have started to stagnate. We have improved diagnostic tools, screening programmes and treatments for CVDs and cancers the last 30 years; however, many people live with residual conditions as well as multiple comorbidities that can have serious economic repercussions for health care and society.

While being cautious in inferring these findings to working ability and pension age, it is important to understand that the rate of some of the conditions that often impact working ability, have not improved much in recent years, and that a longer working life therefore cannot be expected for everyone. These results also give additional insight into the debate on compression of mortality vs. compression of morbidity,^3^ especially when looking at changes in burden of disease in Western and Eastern European regions. There is strong evidence that no compression of morbidity has taken place overall in Europe. A big difference was seen in the unadjusted YLD rates for Western Europe, especially for females since 2010 who have experienced increasing YLDs. With new data emerging on COVID-19, which is not included in this study, we can likely anticipate an increase not only in YLLs but also in YLDs among already vulnerable older Europeans with chronic comorbidities.^20^

### Strengths and limitations

As we used data from the GBD 2019 study, well-established limitations of the GBD methodology also apply here. One limitation is that we reported YLDs, YLLs and DALYs by Level 2 cause of disease categories rather than the more detailed Level 3 or 4 cause of disease categories and therefore this may hide more specific diseases relevant for ageing. Also, we were not able to quantify UI because we did not have access to the posterior samples (1000 draws) of YLL, YLD or DALY rates stratified by age, sex, location and year from the GBD 2019 study. Furthermore, our study only presents insights into patterns within the three European regions, meaning that the findings are unlikely to be generalizable to each country within a given region.

## Supplementary data

Supplementary data are available at *EURPUB* online.

## Supplementary Material

ckac160_Supplementary_Data

## Data Availability

Publicly available datasets were analyzed in this study. The data can be found here: http://ghdx.healthdata.org/gbd-results-tool. Unique, differing and complex trends in the burden of disease for older adults are observed across European regions over the last 30 years. The decline in disease burden in Europe was mainly driven by a reduction in YLL. The rate of YLDs has not declined in Europe and only small improvements have taken place. It is necessary to focus on preventative strategies to target drivers of burden of disease and better manage NCDs in Europe.
